# 3-(Benzo[*d*]thia­zol-2-yl)-2*H*-chromen-2-one

**DOI:** 10.1107/S2414314622003327

**Published:** 2022-03-31

**Authors:** Amira E. M. Abdallah, Galal H. Elgemeie, Peter G. Jones

**Affiliations:** a Chemistry Department, Faculty of Science, Helwan University, Cairo, Egypt; b Institut für Anorganische und Analytische Chemie, Technische Universität Braunschweig, Hagenring 30, D-38106 Braunschweig, Germany; Università di Parma, Italy

**Keywords:** benzo­thia­zol, coumarin, chromene, crystal structure

## Abstract

In the title compound, C_16_H_9_NO_2_S, the inter­planar angle is 6.47 (6)°. An intra­molecular S⋯O=C contact of 2.727 (2) Å is observed. The packing is determined by several types of weak inter­action (‘weak’ hydrogen bonds, S⋯S contacts and π–π stacking).

## Abstract 2

The inter­planar angle is 6.47 (6)°, with an associated intra­molecular S⋯O= C contact. The packing involves various secondary inter­actions.

## Graphical abstract

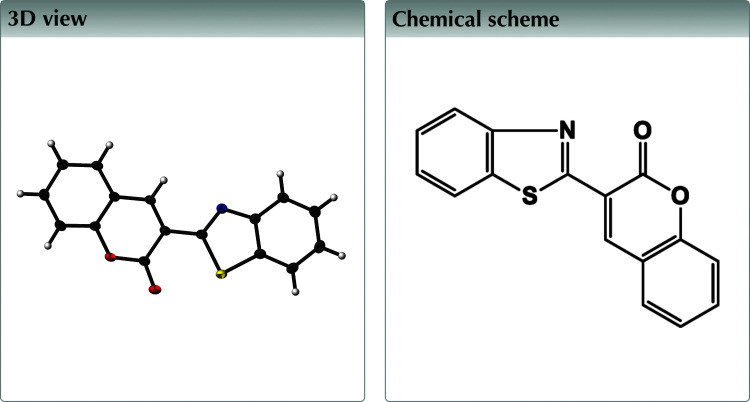

## Structure description

Coumarin (chromen-2-one) derivatives represent a significant class of organic heterocycles; they can be found in various natural or synthetic drug compounds and display a variety of biological activities (Curini *et al.*, 2006 ▸), the most noticeable of which are photobiological properties upon irradiation with UV light. Thus, many coumarin derivatives are effective photosensitizers with valuable applications in medicine (Bansal *et al.*, 2013 ▸). Because of these photochemical characteristics, together with their practical stability, ease of synthesis and good solubility, coumarins have been widely explored as solar energy collectors, charge-transfer agents and non-linear optical materials for photonic and electronic applications (Kim *et al.*, 2011 ▸). Coumarins are one of the most broadly investigated and commercially important classes of organic fluorescent materials. Coumarin dyes fluoresce in the blue–green spectroscopic region and are commonly used in daylight fluorescent pigments, fluorescent probes, and as tunable dye lasers or organic light-emitting diodes (Christie & Lui, 2000 ▸). The emission intensity of coumarin chromophores strongly depends on the nature and position of their substituents (Żamojć *et al.*, 2014 ▸).

Benzo­thia­zole derivatives also exhibit strong luminescence in solution and in the solid state. Mol­ecules that incorporate benzo­thia­zoles have attracted considerable research inter­est in the field of organic light-emitting diodes because of their unique electro-optical properties (Wang *et al.*, 2010 ▸). Recently, we have synthesized some benzo­thia­zoles (Azzam *et al.*, 2017*a* ▸,*b* ▸, 2020*a* ▸,*b* ▸,*c* ▸, 2021 ▸; Elgemeie *et al.*, 2000*a* ▸,*b* ▸) and coumarin derivatives (Elgemeie, 1989 ▸; Elgemeie & Elghandour, 1990 ▸; Elgemeie *et al.*, 2015 ▸).

**Figure 1:**
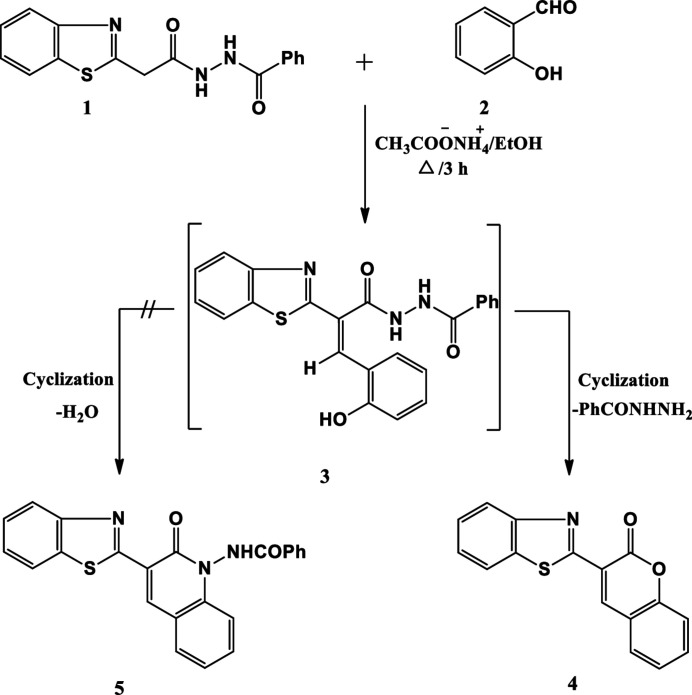
Reaction scheme.

As a continuation of our research inter­est in exploiting new coumarin and benzo­thia­zole derivatives for light-emitting materials, we describe here the synthesis and characterization of a benzo­thia­zole-based coumarin compound. The reaction of *N*-[2-(benzo[*d*]thia­zol-2-yl)acet­yl]benzohydrazide (**1**) with salicylaldehde (**2**) was investigated. This gave a product whose mass spectrum was not consistent with the proposed structure *N*-(3-(benzo[*d*]thia­zol-2-yl)-2-oxoquinolin-1(2*H*)-yl)benzamide (**5**). Therefore the X-ray crystal structure was determined, revealing that 3-(benzo[*d*]thia­zol-2-yl)-2*H*-chromen-2-one (**4**) is the sole product in the solid state (Fig. 1 ▸). The formation of (**4**) is assumed to proceed *via* initial formation of adduct (**3**) and elimination of benzohydrazide rather than water.

**Figure 2:**
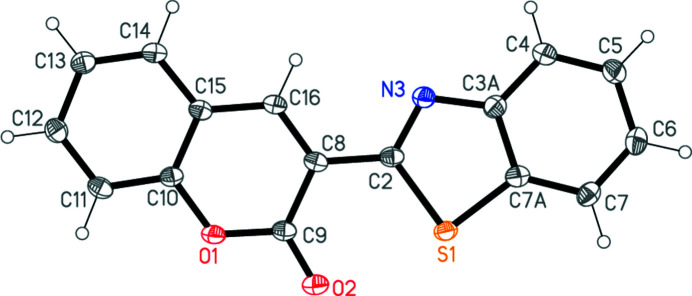
The mol­ecule of **4** in the crystal. Ellipsoids represent 50% probability levels.

The structure of **4** is shown in Fig. 2 ▸. Mol­ecular dimensions may be regarded as normal; a brief selection is presented in Table 1 ▸. The ring systems are essentially planar, with r.m.s. deviations of 0.012 Å for the benzo­thia­zole and 0.006 Å for the chromene (including the exocyclic oxygen atom); the inter­planar angle is 6.47 (6)°. The intra­molecular contact distance S1⋯O2 is 2.727 (2) Å.

**Figure 3:**
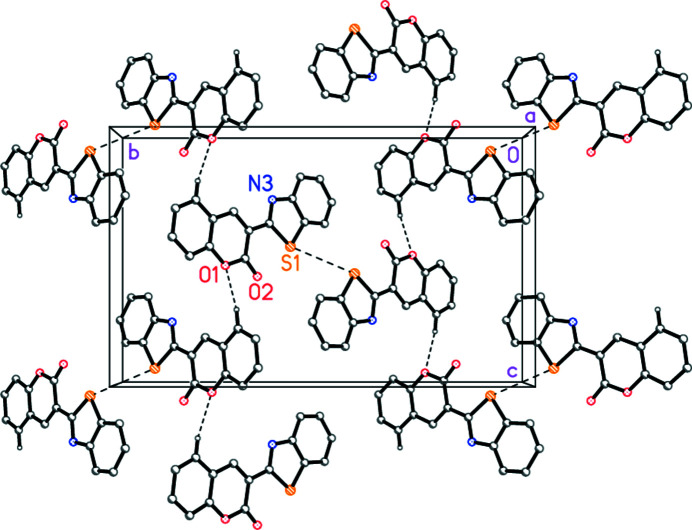
Crystal packing of **4** viewed parallel to the short *a* axis. Dashed lines indicate ‘weak’ hydrogen bonds or S⋯S contacts. Hydrogen atoms not involved in hydrogen bonding are omitted.

There are three short inter­molecular H⋯O/H⋯N contacts (Table 2 ▸), the shortest of which could reasonably be regarded as a ‘weak’ hydrogen bond. Together with an S1⋯S1 contact [3.626 (1) Å, operator −*x*, 1 − *y*, 1 − *z*], this links the mol­ecules to form corrugated sheets lying parallel to the *bc* plane (Fig. 3 ▸). However, this is only one way of considering the packing. The rings also display short contacts between their centroids *via x-*axis translation [thia­zole⋯(benzo­thia­zole benzo) 3.6187 (13) Å, thia­zole⋯(coumarin heterocycle) 3.5344 (12) Å, (coumarin heterocycle)⋯(coumarin benzo) 3.4148 (12) Å], although the stacking is somewhat offset. Viewed parallel to the *c* axis, the rings are seen edge-on in a herringbone pattern.

A search of the Cambridge Database (Version 2021.3.0; Groom *et al.*, 2016 ▸) for purely organic coumarin derivatives gave 1030 hits with 1299 individual mol­ecules. The mean bond lengths of the coumarin heterocycle (referred to the numbering of **4**) are: C15—C16 = 1.442 (15), C16—C8 =1.356 (19), C8—C9 =1.448 (18), C9—O1 =1.375 (14) and O1—C10 =1.378 (11) Å, and the values in **4** (Table 1 ▸) are consistent with these mean values. A more specialized search revealed four compounds with simple coumarin derivatives linked to benzo[*d*]thia­zol in the same way as in **4**: DARPIX (Ezeh & Harrop, 2012 ▸), VIVWEF and VIWDOX (Shi *et al.*, 2019 ▸), WINZAU (Jasinski & Paight, 1995 ▸). The first three have been used as fluorescent probes, for the detection of bio­thiols and the evaluation of anti-cancer agents, respectively.

## Synthesis and crystallization

A mixture of *N*-[2-(benzo[*d*]thia­zol-2-yl)acet­yl]benzo­hydra­zide **1** (3.11 g, 0.01 mol), salicyl­aldehyde **2** (1.22 g, 0.01 mol) and ammonium acetate (0.77 g, 0.01 mol) in ethanol (10 mL) was refluxed for 3 h. The precipitate was filtered off and recrystallized from ethanol solution to give pale-yellow crystals in 95% yield, m.p. 501–503 K; IR (KBr, cm^−1^): υ 3048, 3028 (CH-aromatic), 1715 (C=O), 1557 (C=N) and 1602, 1479 (C=C). ^1^H NMR (400 MHz DMSO-*d*_6_) *δ*: 7.46–8.21 (*m*, 8H, 2C_6_H_4_), 9.26 (*s*, 1H, CH-pyran). ^13^C NMR (100 MHz, DMSO-*d_6_*) *δ*: 116.7, 119.2, 119.8, 122.7, 123.0, 125.7, 125.9, 127.2, 130.7, 134.2, 136.4, 142.5, 152.4, 153.8 (aromatic carbons), 159.9 (C=N), 160.1 (C=O). MS (EI): *m*/*z* (%) 281 [*M* ^+^+2] (0.44), 280 [*M* ^+^+1] (0.96), 279 [*M* ^+^] (4.06), 278 [*M* ^+^−1] (0.12), 277 [*M* ^+^−2] (0.17), 105 (100.00), 77 [C_6_H_5_]^+^ (58.06). Analysis: calculated for C_16_H_9_NO_2_S (279.31): C 68.80; H 3.25; N 5.01; S 11.48%. Found: C 68.70; H 3.33; N 5.12; S 11.60%.

The sample consisted of a mass of long, extremely fine needles with an overall matt appearance. Careful separation and cutting provided a single crystal, which proved to be measurable using a high-intensity X-ray source.

## Refinement

Crystal data, data collection and structure refinement details are summarized in Table 3 ▸.

## Supplementary Material

Crystal structure: contains datablock(s) I, global. DOI: 10.1107/S2414314622003327/xi4003sup1.cif

Structure factors: contains datablock(s) I. DOI: 10.1107/S2414314622003327/xi4003Isup2.hkl

Supporting information file. DOI: 10.1107/S2414314622003327/xi4003Isup3.cml

CCDC reference: 2161632

Additional supporting information:  crystallographic information; 3D view; checkCIF report

## Figures and Tables

**Table 1:** Selected geometric parameters (Å, °)

S1—C7*A*	1.733 (2)	C8—C16	1.357 (3)
S1—C2	1.758 (2)	C8—C9	1.470 (3)
C2—N3	1.308 (3)	C9—O1	1.365 (3)
N3—C3*A*	1.380 (3)	C10—O1	1.379 (2)
C3*A*—C7*A*	1.411 (3)	C15—C16	1.434 (3)
			
C7*A*—S1—C2	88.87 (10)	N3—C3*A*—C7*A*	115.15 (19)
N3—C2—S1	115.63 (16)	C3*A*—C7*A*—S1	109.57 (16)
C2—N3—C3*A*	110.75 (18)		

**Table 2:** Hydrogen-bond geometry (Å, °)

*D*—H⋯*A*	*D*—H	H⋯*A*	*D*⋯*A*	*D*—H⋯*A*
C14—H14⋯O1^i^	0.95	2.46	3.383 (3)	163
C11—H11⋯N3^ii^	0.95	2.63	3.523 (3)	156
C13—H13⋯O2^iii^	0.95	2.65	3.314 (3)	127

Symmetry codes: (i) 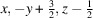 ; (ii) 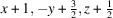 ; (iii) 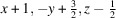 .

**Table 3:** Experimental details

Crystal data	
Chemical formula	C_16_H_9_NO_2_S
*M*_r_	279.30
Crystal system, space group	Monoclinic, *P*2_1_/*c*
Temperature (K)	100
*a*, *b*, *c* (Å)	4.60717 (11), 20.7275 (5), 12.6444 (3)
β (°)	91.911 (2)
*V* (Å^3^)	1206.81 (5)
*Z*	4
Radiation type	Cu *K*α
μ (mm^−1^)	2.39
Crystal size (mm)	0.04 × 0.01 × 0.01

Data collection	
Diffractometer	XtaLAB Synergy
Absorption correction	Multi-scan (*CrysAlis PRO*; Rigaku OD, 2021 ▸)
*T*_min_, *T*_max_	0.696, 1.000
No. of measured, independent and observed [*I* > 2σ(*I*)] reflections	49602, 2547, 2528
*R*_int_	0.068
(sin θ/λ)_max_ (Å^−1^)	0.634

Refinement	
*R*[*F* ^2^ > 2σ(*F* ^2^)], *wR*(*F* ^2^), *S*	0.049, 0.120, 1.25
No. of reflections	2547
No. of parameters	181
H-atom treatment	H-atom parameters constrained
Δρ_max_, Δρ_min_ (e Å^−3^)	0.45, −0.43

Computer programs: *CrysAlis PRO* (Rigaku OD, 2021 ▸), *SHELXT* (Sheldrick, 2015*b* ▸), *SHELXL2018/3* (Sheldrick, 2015*a* ▸) and *XP* (Siemens, 1994 ▸).

## full crystallographic data

### Crystal data

**Table:**

C_16_H_9_NO_2_S	*F*(000) = 576
*M**_r_* = 279.30	*D*_x_ = 1.537 Mg m^−^^3^
Monoclinic, *P*2_1_/*c*	Cu *K*α radiation, λ = 1.54184 Å
*a* = 4.60717 (11) Å	Cell parameters from 20622 reflections
*b* = 20.7275 (5) Å	θ = 4.1–76.8°
*c* = 12.6444 (3) Å	µ = 2.38 mm^−^^1^
β = 91.911 (2)°	*T* = 100 K
*V* = 1206.81 (5) Å^3^	Needle, pale yellow
*Z* = 4	0.04 × 0.01 × 0.01 mm

### Data collection

**Table:**

XtaLAB Synergy diffractometer	2547 independent reflections
Radiation source: micro-focus sealed X-ray tube	2528 reflections with *I* > 2σ(*I*)
Detector resolution: 10.0000 pixels mm^-1^	*R*_int_ = 0.068
ω scans	θ_max_ = 77.9°, θ_min_ = 4.1°
Absorption correction: multi-scan (CrysAlisPro; Rigaku OD, 2021)	*h* = −5→5
*T*_min_ = 0.696, *T*_max_ = 1.000	*k* = −24→26
49602 measured reflections	*l* = −15→15

### Refinement

**Table:**

Refinement on *F*^2^	Primary atom site location: dual
Least-squares matrix: full	Hydrogen site location: inferred from neighbouring sites
*R*[*F*^2^ > 2σ(*F*^2^)] = 0.049	H-atom parameters constrained
*wR*(*F*^2^) = 0.120	*w* = 1/[σ^2^(*F*_o_^2^) + (0.0479*P*)^2^ + 1.2289*P*] where *P* = (*F*_o_^2^ + 2*F*_c_^2^)/3
*S* = 1.25	(Δ/σ)_max_ = 0.001
2547 reflections	Δρ_max_ = 0.45 e Å^−^^3^
181 parameters	Δρ_min_ = −0.43 e Å^−^^3^
0 restraints	

### Special details

**Table:**

Geometry. All esds (except the esd in the dihedral angle between two l.s. planes) are estimated using the full covariance matrix. The cell esds are taken into account individually in the estimation of esds in distances, angles and torsion angles; correlations between esds in cell parameters are only used when they are defined by crystal symmetry. An approximate (isotropic) treatment of cell esds is used for estimating esds involving l.s. planes. Least-squares planes (x,y,z in crystal coordinates) and deviations from them (* indicates atom used to define plane) - 3.3898 (0.0014) x + 13.7552 (0.0080) y + 2.0177 (0.0070) z = 8.9676 (0.0037) * 0.0227 (0.0011) S1 * 0.0005 (0.0015) C2 * -0.0077 (0.0015) N3 * -0.0083 (0.0019) C3A * 0.0016 (0.0017) C4 * 0.0176 (0.0017) C5 * 0.0019 (0.0017) C6 * -0.0169 (0.0016) C7 * -0.0115 (0.0018) C7A Rms deviation of fitted atoms = 0.0124 - 3.2516 (0.0012) x + 14.6654 (0.0053) y + 0.7477 (0.0068) z = 9.1325 (0.0065) Angle to previous plane (with approximate esd) = 6.467 ( 0.060 ) * -0.0054 (0.0017) C8 * -0.0016 (0.0018) C9 * 0.0001 (0.0019) C10 * -0.0072 (0.0017) C11 * 0.0011 (0.0017) C12 * -0.0035 (0.0017) C13 * -0.0017 (0.0017) C14 * 0.0079 (0.0019) C15 * 0.0022 (0.0016) C16 * 0.0136 (0.0014) O1 * -0.0054 (0.0014) O2 Rms deviation of fitted atoms = 0.0059#======================================================================Short contact: 3.6259 (0.0010) S1 - S1_$4 Operator $4: -x, 1-y, 1-z
Refinement. The hydrogen atoms were included using a riding model starting from calculated positions (C—H_aromatic_ 0.95 Å). The *U*(H) values were fixed at 1.2 times the equivalent *U*_iso_ value of the parent carbon atoms.

### Fractional atomic coordinates and isotropic or equivalent isotropic displacement parameters (Å^2^)

**Table:**

	*x*	*y*	*z*	*U*_iso_*/*U*_eq_	
S1	−0.03347 (11)	0.58024 (2)	0.44385 (4)	0.01871 (16)	
C2	0.1290 (5)	0.63202 (10)	0.35289 (16)	0.0179 (4)	
N3	0.0429 (4)	0.62471 (9)	0.25396 (14)	0.0190 (4)	
C3A	−0.1604 (5)	0.57600 (10)	0.24410 (17)	0.0197 (4)	
C4	−0.3012 (5)	0.55593 (11)	0.14936 (17)	0.0227 (5)	
H4	−0.257833	0.575871	0.084112	0.027*	
C5	−0.5025 (5)	0.50703 (11)	0.15244 (18)	0.0238 (5)	
H5	−0.600561	0.493791	0.088821	0.029*	
C6	−0.5657 (5)	0.47627 (11)	0.24818 (18)	0.0230 (5)	
H6	−0.704244	0.442317	0.248094	0.028*	
C7	−0.4295 (5)	0.49470 (10)	0.34205 (18)	0.0208 (4)	
H7	−0.471183	0.473769	0.406635	0.025*	
C7A	−0.2284 (5)	0.54501 (10)	0.33950 (16)	0.0189 (4)	
C8	0.3479 (4)	0.67984 (10)	0.38551 (16)	0.0174 (4)	
C9	0.4134 (5)	0.68882 (10)	0.49914 (16)	0.0186 (4)	
C10	0.7662 (5)	0.76940 (10)	0.45552 (16)	0.0174 (4)	
C11	0.9720 (5)	0.81249 (10)	0.49547 (16)	0.0200 (4)	
H11	1.009311	0.816281	0.569554	0.024*	
C12	1.1223 (5)	0.84998 (10)	0.42467 (17)	0.0208 (4)	
H12	1.262170	0.880282	0.450509	0.025*	
C13	1.0693 (5)	0.84351 (10)	0.31510 (17)	0.0202 (4)	
H13	1.174863	0.869054	0.267249	0.024*	
C14	0.8647 (5)	0.80021 (10)	0.27695 (16)	0.0191 (4)	
H14	0.830301	0.796009	0.202773	0.023*	
C15	0.7059 (4)	0.76208 (10)	0.34688 (16)	0.0173 (4)	
C16	0.4906 (4)	0.71565 (10)	0.31393 (16)	0.0173 (4)	
H16	0.447058	0.709837	0.240584	0.021*	
O1	0.6178 (3)	0.73370 (7)	0.52821 (11)	0.0198 (3)	
O2	0.2995 (4)	0.65973 (8)	0.56960 (12)	0.0247 (4)	

### Atomic displacement parameters (Å^2^)

**Table:**

	*U*^11^	*U*^22^	*U*^33^	*U*^12^	*U*^13^	*U*^23^
S1	0.0214 (3)	0.0199 (3)	0.0150 (3)	−0.00055 (19)	0.00235 (18)	0.00232 (18)
C2	0.0205 (10)	0.0179 (10)	0.0156 (9)	0.0036 (8)	0.0022 (7)	0.0008 (7)
N3	0.0211 (9)	0.0194 (9)	0.0166 (8)	−0.0004 (7)	0.0017 (7)	−0.0005 (7)
C3A	0.0209 (10)	0.0180 (10)	0.0203 (10)	0.0004 (8)	0.0033 (8)	−0.0002 (8)
C4	0.0265 (11)	0.0241 (11)	0.0175 (10)	−0.0013 (9)	0.0009 (8)	−0.0015 (8)
C5	0.0268 (11)	0.0234 (11)	0.0213 (11)	−0.0010 (9)	0.0017 (8)	−0.0048 (8)
C6	0.0212 (11)	0.0182 (10)	0.0300 (12)	−0.0016 (8)	0.0042 (9)	−0.0026 (9)
C7	0.0212 (10)	0.0180 (10)	0.0237 (11)	0.0006 (8)	0.0059 (8)	0.0014 (8)
C7A	0.0210 (10)	0.0180 (10)	0.0178 (10)	0.0034 (8)	0.0026 (8)	0.0011 (8)
C8	0.0190 (10)	0.0190 (10)	0.0142 (9)	0.0034 (8)	0.0002 (7)	−0.0005 (7)
C9	0.0207 (10)	0.0204 (10)	0.0149 (9)	0.0024 (8)	0.0021 (8)	−0.0003 (8)
C10	0.0200 (10)	0.0171 (10)	0.0153 (9)	0.0017 (8)	0.0026 (7)	0.0004 (7)
C11	0.0236 (11)	0.0206 (10)	0.0158 (9)	0.0034 (8)	−0.0005 (8)	−0.0034 (8)
C12	0.0217 (10)	0.0191 (10)	0.0214 (10)	0.0002 (8)	−0.0010 (8)	−0.0023 (8)
C13	0.0221 (10)	0.0193 (10)	0.0194 (10)	0.0017 (8)	0.0024 (8)	0.0013 (8)
C14	0.0229 (10)	0.0199 (10)	0.0145 (9)	0.0021 (8)	0.0012 (8)	0.0008 (8)
C15	0.0201 (10)	0.0169 (10)	0.0147 (10)	0.0027 (8)	−0.0004 (7)	0.0001 (7)
C16	0.0192 (10)	0.0197 (10)	0.0130 (9)	0.0026 (8)	0.0007 (7)	−0.0002 (7)
O1	0.0242 (8)	0.0227 (8)	0.0125 (7)	−0.0010 (6)	0.0010 (6)	0.0005 (6)
O2	0.0303 (8)	0.0281 (8)	0.0157 (7)	−0.0034 (7)	0.0030 (6)	0.0022 (6)

### Geometric parameters (Å, º)

**Table:**

S1—C7A	1.733 (2)	C10—C11	1.385 (3)
S1—C2	1.758 (2)	C10—C15	1.401 (3)
C2—N3	1.308 (3)	C11—C12	1.388 (3)
C2—C8	1.464 (3)	C12—C13	1.405 (3)
N3—C3A	1.380 (3)	C13—C14	1.377 (3)
C3A—C4	1.406 (3)	C14—C15	1.409 (3)
C3A—C7A	1.411 (3)	C15—C16	1.434 (3)
C4—C5	1.375 (3)	C4—H4	0.9500
C5—C6	1.407 (3)	C5—H5	0.9500
C6—C7	1.378 (3)	C6—H6	0.9500
C7—C7A	1.396 (3)	C7—H7	0.9500
C8—C16	1.357 (3)	C11—H11	0.9500
C8—C9	1.470 (3)	C12—H12	0.9500
C9—O2	1.210 (3)	C13—H13	0.9500
C9—O1	1.365 (3)	C14—H14	0.9500
C10—O1	1.379 (2)	C16—H16	0.9500
			
C7A—S1—C2	88.87 (10)	C14—C13—C12	120.1 (2)
N3—C2—C8	122.13 (19)	C13—C14—C15	120.63 (19)
N3—C2—S1	115.63 (16)	C10—C15—C14	117.67 (19)
C8—C2—S1	122.24 (15)	C10—C15—C16	118.07 (19)
C2—N3—C3A	110.75 (18)	C14—C15—C16	124.25 (19)
N3—C3A—C4	125.9 (2)	C8—C16—C15	121.26 (19)
N3—C3A—C7A	115.15 (19)	C9—O1—C10	122.61 (16)
C4—C3A—C7A	119.0 (2)	C5—C4—H4	120.5
C5—C4—C3A	119.1 (2)	C3A—C4—H4	120.5
C4—C5—C6	121.2 (2)	C4—C5—H5	119.4
C7—C6—C5	120.9 (2)	C6—C5—H5	119.4
C6—C7—C7A	118.1 (2)	C7—C6—H6	119.5
C7—C7A—C3A	121.7 (2)	C5—C6—H6	119.5
C7—C7A—S1	128.67 (17)	C6—C7—H7	121.0
C3A—C7A—S1	109.57 (16)	C7A—C7—H7	121.0
C16—C8—C2	121.80 (18)	C10—C11—H11	120.8
C16—C8—C9	119.64 (19)	C12—C11—H11	120.8
C2—C8—C9	118.56 (18)	C11—C12—H12	119.7
O2—C9—O1	116.96 (18)	C13—C12—H12	119.7
O2—C9—C8	125.2 (2)	C14—C13—H13	119.9
O1—C9—C8	117.81 (18)	C12—C13—H13	119.9
O1—C10—C11	116.83 (18)	C13—C14—H14	119.7
O1—C10—C15	120.59 (19)	C15—C14—H14	119.7
C11—C10—C15	122.58 (19)	C8—C16—H16	119.4
C10—C11—C12	118.41 (19)	C15—C16—H16	119.4
C11—C12—C13	120.6 (2)		
			
C7A—S1—C2—N3	1.07 (17)	C16—C8—C9—O2	179.9 (2)
C7A—S1—C2—C8	−178.49 (18)	C2—C8—C9—O2	−0.2 (3)
C8—C2—N3—C3A	179.04 (18)	C16—C8—C9—O1	0.2 (3)
S1—C2—N3—C3A	−0.5 (2)	C2—C8—C9—O1	−179.90 (17)
C2—N3—C3A—C4	179.1 (2)	O1—C10—C11—C12	−178.97 (18)
C2—N3—C3A—C7A	−0.5 (3)	C15—C10—C11—C12	0.4 (3)
N3—C3A—C4—C5	−179.1 (2)	C10—C11—C12—C13	−0.9 (3)
C7A—C3A—C4—C5	0.5 (3)	C11—C12—C13—C14	0.7 (3)
C3A—C4—C5—C6	−1.1 (3)	C12—C13—C14—C15	0.1 (3)
C4—C5—C6—C7	0.6 (3)	O1—C10—C15—C14	179.76 (18)
C5—C6—C7—C7A	0.4 (3)	C11—C10—C15—C14	0.5 (3)
C6—C7—C7A—C3A	−1.0 (3)	O1—C10—C15—C16	−1.3 (3)
C6—C7—C7A—S1	177.72 (17)	C11—C10—C15—C16	179.42 (19)
N3—C3A—C7A—C7	−179.79 (19)	C13—C14—C15—C10	−0.7 (3)
C4—C3A—C7A—C7	0.6 (3)	C13—C14—C15—C16	−179.6 (2)
N3—C3A—C7A—S1	1.3 (2)	C2—C8—C16—C15	−179.62 (19)
C4—C3A—C7A—S1	−178.36 (16)	C9—C8—C16—C15	0.3 (3)
C2—S1—C7A—C7	179.9 (2)	C10—C15—C16—C8	0.2 (3)
C2—S1—C7A—C3A	−1.24 (16)	C14—C15—C16—C8	179.1 (2)
N3—C2—C8—C16	−5.4 (3)	O2—C9—O1—C10	179.04 (18)
S1—C2—C8—C16	174.10 (16)	C8—C9—O1—C10	−1.2 (3)
N3—C2—C8—C9	174.66 (19)	C11—C10—O1—C9	−178.83 (18)
S1—C2—C8—C9	−5.8 (3)	C15—C10—O1—C9	1.8 (3)

### Hydrogen-bond geometry (Å, º)

**Table:**

*D*—H···*A*	*D*—H	H···*A*	*D*···*A*	*D*—H···*A*
C14—H14···O1^i^	0.95	2.46	3.383 (3)	163
C11—H11···N3^ii^	0.95	2.63	3.523 (3)	156
C13—H13···O2^iii^	0.95	2.65	3.314 (3)	127

Symmetry codes: (i) *x*, −*y*+3/2, *z*−1/2; (ii) *x*+1, −*y*+3/2, *z*+1/2; (iii) *x*+1, −*y*+3/2, *z*−1/2.
